# Genome sequence analyses show that *Neisseria oralis* is the same species as ‘*Neisseria mucosa* var. *heidelbergensis*’

**DOI:** 10.1099/ijs.0.052431-0

**Published:** 2013-10

**Authors:** Julia S. Bennett, Keith A. Jolley, Martin C. J. Maiden

**Affiliations:** Department of Zoology, University of Oxford, Oxford OX1 3PS, UK

## Abstract

Phylogenies generated from whole genome sequence (WGS) data provide definitive means of bacterial isolate characterization for typing and taxonomy. The species status of strains recently defined with conventional taxonomic approaches as representing *Neisseria oralis* was examined by the analysis of sequences derived from WGS data, specifically: (i) 53 *Neisseria* ribosomal protein subunit (*rps*) genes (ribosomal multi-locus sequence typing, rMLST); and (ii) 246 *Neisseria* core genes (core genome MLST, cgMLST). These data were compared with phylogenies derived from 16S and 23S rRNA gene sequences, demonstrating that the *N. oralis* strains were monophyletic with strains described previously as representing ‘*Neisseria mucosa* var. *heidelbergensis*’ and that this group was of equivalent taxonomic status to other well-described species of the genus *Neisseria*. Phylogenetic analyses also indicated that *Neisseria sicca* and *Neisseria macacae* should be considered the same species as *Neisseria mucosa* and that *Neisseria flavescens* should be considered the same species as *Neisseria subflava*. Analyses using rMLST showed that some strains currently defined as belonging to the genus *Neisseria* were more closely related to species belonging to other genera within the family; however, whole genome analysis of a more comprehensive selection of strains from within the family *Neisseriaceae* would be necessary to confirm this. We suggest that strains previously identified as representing ‘*N. mucosa* var. *heidelbergensis*’ and deposited in culture collections should be renamed *N. oralis*. Finally, one of the strains of *N. oralis* was able to ferment lactose, due to the presence of β-galactosidase and lactose permease genes, a characteristic previously thought to be unique to *Neisseria lactamica*, which therefore cannot be thought of as diagnostic for this species; however, the rMLST and cgMLST analyses confirm that *N. oralis* is most closely related to *N. mucosa*.

In 1987, the report of an ad hoc subcommittee of the International Committee for Systematic Bacteriology (Wayne *et al.*, 1987) recommended that phylogeny should determine bacterial taxonomy and that the complete DNA sequence should be regarded as the standard for determining these phylogenies. This recommendation was reinforced and extended in 2002 by a further ad hoc subcommittee report, which recognized that the extensive structuring of prokaryote diversity can be identified with appropriate molecular techniques and used as a basis for nomenclature. This latter report further identified the sequencing of housekeeping genes as a method of great promise for the development of molecular systematics (Stackebrandt *et al.*, 2002). While many whole genome sequences (WGSs) of bacterial isolates are now available, the analysis of multiple sets of WGS data remains complicated by the diversity of the prokaryotic domains, with few genes shared among all bacteria. Ribosomal multi-locus sequence typing (rMLST) resolves this problem by indexing variation at a set of genes, those encoding the ribosomal protein subunits (the *rps* genes), the great majority of which are found in all bacteria (Jolley *et al.*, 2012a).

The rMLST approach provides more resolution of sequence clusters than 16S or 23S rRNA gene phylogenies and has been applied at the whole domain level (Jolley *et al.*, 2012a) and to examine species and subspecies structuring within the genus *Neisseria* (Bennett *et al.*, 2012; Jolley *et al.*, 2012b). The genus *Neisseria* is an instructive model for the development of novel bacterial characterization techniques as it comprises a number of organisms poorly distinguished by conventional methods including biochemical tests and 16S and 23S rRNA analyses (Harmsen *et al.*, 2001; Teng *et al.*, 2011; Zhu *et al.*, 2003). Different species of the genus *Neisseria* nevertheless exhibit distinct and stable differences in their phenotypes, particularly as regards their pathogenicity (Maiden, 2008). The relationships among members of the genus have been shown to be well resolved by rMLST, validated with an analysis of 246 core genes (core genome MLST, cgMLST) (Bennett *et al.*, 2012).

A recent polyphasic analysis of seven isolates of Gram-negative cocci, collected from pathological clinical samples and healthy subgingival plaque from patients in the USA, suggested that these isolates represented a novel species of the genus *Neisseria* (*Neisseria oralis*), most closely related to *Neisseria lactamica*. This novel species was defined using a number of conventional methods for species characterization, including: 16S rRNA and 23S rRNA gene sequence similarity; DNA–DNA hybridization; cellular fatty acid analysis; and several phenotypic analyses (Wolfgang *et al.*, 2013). To compare this novel species with other members of the family *Neisseriaceae*, and establish their relationships at the genomic level, the genome sequence for *N. oralis* F0314 was obtained from the Integrated Microbial Genomes database (Markowitz *et al.*, 2010) and uploaded to the PubMLST *Neisseria* database: http://pubmlst.org/neisseria/ (Jolley & Maiden, 2010; Jolley & Maiden, 2013). Here it was directly compared with genome sequences obtained from 51 *Neisseria* isolates, including the type strains of 18 recognized species of the genus, and a strain deposited as the ‘type strain’ of ‘*Neisseria mucosa* var. *heidelbergensis*’ (Berger, 1971), in the American type Culture Collection as ATCC 25999 (CCUG 26878). In addition, nucleotide sequences for the 16S rRNA and 23S rRNA fragments for *N. oralis* (Wolfgang *et al.*, 2013) were uploaded to the PubMLST *Neisseria* database. All nucleotide sequences used in this study are publicly available, either from http://pubmlst.org/neisseria/ for the *Neisseria* loci or from http://pubmlst.org/rmlst/ for the *Eikenella corrodens*, *Kingella oralis* and *Simonsiella muelleri* rMLST loci (Table S1 available in IJSEM Online). Nucleotide sequences were concatenated using the BIGSdb platform on PubMLST (Jolley & Maiden, 2010) and aligned using Muscle version 3.7 (Edgar, 2004). Phylogenetic analyses were undertaken using mega 5.1 (Tamura *et al.*, 2011) and SplitsTree 4 (Huson & Bryant, 2006), with genetic distances determined according to the Kimura two-parameter model (Kimura, 1980) and phylogenies reconstructed with the neighbour-joining, minimum-evolution and neighbour-net (Bryant & Moulton, 2004) methods. Estimates of evolutionary divergence between sequences were undertaken with mega 5.1 (Tamura *et al.*, 2011), using the Kimura two-parameter model (Kimura, 1980). All ambiguous positions were removed for each pairwise sequence comparison and bootstrap values were based on 1000 replications. Some genes were not identified in some isolates and in a small number of cases gene sequences were incomplete; however, this did not affect the topologies of the phylogenies reconstructed, and different combinations of concatenated sequences gave indistinguishable results. The use of different substitution models or tree-building methods also had no effect on the phylogenetic relationships when concatenated core gene sequences were examined.

Each of the gene sets from the type strains of each of the taxa were compared with a genome sequence for the type strain of *Neisseria gonorrhoeae*, the type species for the genus. This demonstrated the high similarity of the 16S and 23S rRNA genes across the genus, when compared with the diversity present in the rMLST and core gene sets, which also included many more nucleotides (Table 1). For example, the level of similarity to *N. gonorrhoeae* among 16S rRNA gene sequences ranged from 98.61 % for *Neisseria meningitidis* to 94.47 % for *Neisseria elongata subsp. e**longata*, and among 23S rRNA sequences it ranged from 98.95 % for *N. meningitidis* to 94.37 % for *Neisseria bacilliformis*. Three species had >98 % 16S rRNA gene sequence similarity to the type species and 14 species had >98 % 23S rRNA gene sequence similarity. In contrast, the similarity to *N. gonorrhoeae* among the concatenated rMLST sequences ranged from 97.37 % for *N. meningitidis* to 78.72 % for *N. bacilliformis*, and among the concatenated cgMLST sequences it ranged from 95.34 % for *N. meningitidis* to 70.53 % for *Neisseria shayeganii*. No species had >98 % similarity to *N. gonorrhoeae* when both sets of concatenated core genes were examined. Phylogenies reconstructed from the 16S and 23S rRNA gene sequences (Figs S1 and S2, respectively) were incongruent and did not cluster the taxa consistently regardless of the tree-building method used, due to the weak phylogenetic signal; however, both of these phylogenies clustered ‘*N. mucosa* var. *heidelbergensis*’ strains with *N. oralis* strains, with 100 % bootstrap support for the 16S rRNA gene sequence cluster. For both 16S and 23S rRNA gene phylogenies, the two species were indistinguishable; indeed, isolate CCUG 804 (Berger M33), defined originally as representing *Neisseria mucosa* but identified as representing ‘*N. mucosa* var. *heidelbergensis*’ using rMLST, had an identical 16S rRNA gene sequence to the type strain of *N. oralis* (6332^T^). As noted by Wolfgang *et al.* (2013), the 16S rRNA gene phylogeny indicated that the most closely related species to these organisms was *N. lactamica*, but this was not supported by the 23S rRNA gene phylogeny.

**Table 1.** Calculations of nucleotide sequence divergence Percentage sequence similarity to the type species *N. gonorrhoeae* using 16S and 23S rRNA genes and concatenated rMLST and cgMLST genes. In the absence of a genome sequence for the type strain of *N. gonorrhoeae*, the genome sequence of isolate FA1090 was used.

Isolate	Published species designation (suggested designation)	16S rRNA (1537 bp)	23S rRNA (2969 bp)	rMLST (21 629 bp)	cgMLST (191 474 bp)
FAM18	*N. meningitidis*	98.61	98.95	97.37	95.34
ATCC 43768^T^	*N. polysaccharea*	98.54	98.53	96.59	94.36
ATCC 23970^T^	*N. lactamica*	96.31	98.46	92.81	92.99
ATCC 14685^T^	*N. cinerea*	98.14	98.57	91.66	90.57
ATCC 29256^T^	*N. sicca* (*N. mucosa*)	96.31	98.35	90.65	87.16
ATCC 19696^T^	*N. mucosa*	96.51	98.53	90.67	87.09
CCUG 4145^T^	*N. macacae* (*N. mucosa*)	96.37	98.53	90.70	86.95
CCUG 17913^T^	*N. flavescens* (*N. subflava*)	97.39	97.89	89.87	85.13
CCUG 23930^T^	*N. subflava*	96.85	98.10	89.87	84.75
F0314	*N. oralis*	94.85	98.30	89.30	84.34
CCUG 26878^T^	‘*N. mucosa* *var. heidelbergensis*’	94.70	98.07	89.17	84.15
CCUG 808^T^	*N. animalis*	96.31	95.27	86.86	78.70
CCUG 53898^T^	*N. dentiae*	95.69	94.44	83.90	76.31
ATCC 29315^T^	*N. elongata subsp. glyc**olytica*	94.53	98.25	83.39	75.69
CCUG 4554	‘*N. elongate* subsp. *intermedia*’	94.47	98.35	83.15	75.65
CCUG 30802^T^	*N. elongata subsp. nitror**educens*	94.61	98.35	83.10	75.52
CCUG 2043^T^	*N. elongata subsp. e**longata*	94.47	98.35	82.93	75.51
CCUG 50858^T^	*N. bacilliformis*	95.62	94.37	78.72	73.57
CCUG 4007^T^	*N. weaveri*	96.64	94.94	81.48	72.54
CCUG 56775^T^	*N. canis*	95.40	95.05	79.30	71.83
9715^T^	*N. wadsworthii*	94.63	94.83	79.06	71.06
871^T^	*N. shayeganii*	95.27	94.42	78.74	70.53
ATCC 51147^T^	*K. oralis*	93.82	93.89	78.37	−
ATCC 29453^T^	*S. muelleri*	93.07	94.45	77.86	−
ATCC 23834^T^	*E. corrodens*	93.47	93.94	76.02	−

The phylogenies reconstructed from concatenated rMLST (Fig. 1) and cgMLST (Fig. 2) loci produced congruent relationships that were consistent with current *Neisseria* species groupings, with only minor reassignment of strains necessary (Bennett *et al.*, 2012). These phylogenies, as well as a nucleotide similarity of 98.77 % among the strains, confirmed that *N. oralis* is the same species as ‘*N. mucosa* var. *heidelbergensis*’, which has been shown previously to be distinct taxonomically (Bennett *et al.*, 2012), with the suggested name ‘*Neisseria* *heidelbergensis*’. These phylogenies and the percentage similarities of the type strains also indicated that *Neisseria flavescens* should be considered the same species as *Neisseria subflava* (98.76 % similarity), and that *Neisseria macacae* and *Neisseria sicca* should be considered the same species as *N. mucosa* (98.66 and 98.47 % similarity, respectively). These suggested species designations are based on historical precedence: *N. subflava* was described by Flügge in 1886 (Tonjum, 2005), whereas *N. flavescens* was described in 1930 (Branham, 1930); *N. mucosa* was described by von Lingelsheim in 1906, whereas *N. sicca* was described by von Lingelsheim in 1908 (Tonjum, 2005), and *N. macacae* was described in 1983 (Vedros *et al.*, 1983). These phylogenies further confirmed the close relationships among *N. meningitidis*, *N. gonorrhoeae*, *Neisseria polysaccharea* and *N. lactamica*, supporting DNA–DNA hybridization data (Guibourdenche *et al.*, 1986).

**Fig. 1.**
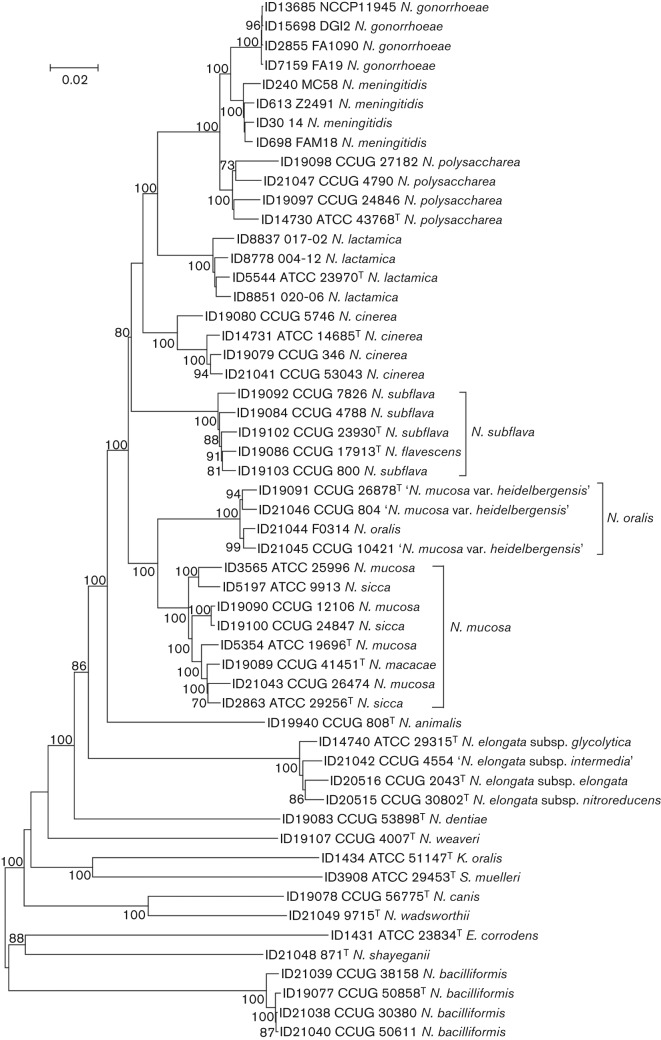
Neighbour-joining tree reconstructed from concatenated ribosomal protein gene sequences. Type strains are indicated with superscript T. Only bootstrap values of 70 % or greater are shown. Suggested species reclassifications are indicated by brackets. Bar, 0.02 substitutions per nucleotide position. ID numbers are the strain identifiers used in the PubMLST *Neisseria* database (http://pubmlst.org/neisseria/) or the rMLST database (http://pubmlst.org/rmlst/).

**Fig. 2.**
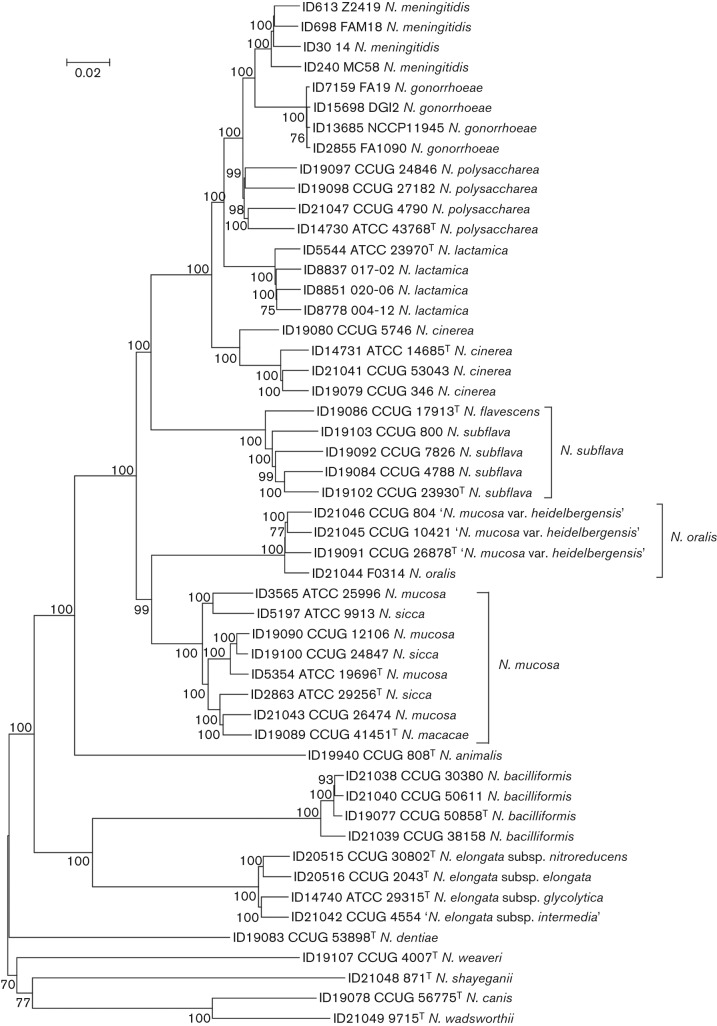
Neighbour-joining tree reconstructed from 246 concatenated core gene sequences. Type strains are indicated with superscript T. Only bootstrap values of 70 % or greater are shown. Suggested species reclassifications are indicated by brackets. Bar, 0.02 substitutions per nucleotide position. ID numbers are the strain identifiers used in the PubMLST *Neisseria* database (http://pubmlst.org/neisseria/).

It has been suggested on the basis of 16S rRNA gene phylogenies that two novel species, *Neisseria wadsworthii* and *N. shayeganii*, are members of a clade that includes *Neisseria dentiae*, *N. bacilliformis* and *Neisseria canis* (Wolfgang *et al.*, 2011). However, both the rMLST (Fig. 1) and the cgMLST (Fig. 2) phylogenies indicated that *N. shayeganii* and *N. wadsworthii* are distinct, distantly related species, with *N. wadsworthii* most closely related to *N. canis*. Calculations of nucleotide sequence divergence using the concatenated rMLST sequences show that *N. shayeganii* and *N. wadsworthii* share 78.74 % similarity and *N. wadsworthii* and *N. canis* share 88.57 % similarity and the closest species to *N. shayeganii* is *N. dentiae*, sharing 80.43 % similarity. Comparisons of nucleotide sequences from *N. gonorrhoeae* with sequences from other species within the family *Neisseriaceae* show that *N. wadsworthii* and *N. shayeganii* are among the species most distantly related to the type species (Table 1). Finally, the rMLST phylogeny demonstrated the close relationship of strains currently assigned to different genera (*K. oralis*, *S. muelleri* and *E. corrodens*) within the family *Neisseriaceae* to species assigned to the genus *Neisseria*, indicating that either these species should be included within the genus *Neisseria* or some species currently defined within the genus *Neisseria* should be reassigned to other genera. For example, a comparison of the concatenated rMLST sequences from the species most distantly related to *N. gonorrhoeae* shows that *N. wadsworthii* is more closely related to *S. muelleri* (77.52 % similarity) and *K. oralis* (76.80 % similarity) than to *N. bacilliformis* (75.98 % similarity), and that *N. shayeganii* is more closely related to *E. corrodens* (77.49 % similarity) than to *Neisseria weaveri* (77.34 % similarity) and *N. bacilliformis* (77.22 % similarity). Whole genome analysis of a more comprehensive selection of strains from within the family *Neisseriaceae* would be necessary to clarify these relationships.

All the phylogenetic reconstructions demonstrated that strains described as representing *N. oralis* (Wolfgang *et al.*, 2013) were monophyletic with strains previously named ‘*N. mucosa* var. *heidelbergensis*’ (Berger, 1971). This group was most closely related to *N. mucosa* although it is distinct from it (Figs 1 and 2), which was inconsistent with the findings that *N. oralis* is a novel species closely related to *N. lactamica* (Wolfgang *et al.*, 2013); however, the relationship to *N. lactamica* was largely suggested on the basis of 16S rRNA gene sequence similarity, which is known to be an unreliable indicator of relationships within the genus (Bennett *et al.*, 2012). Strains belonging to *N. oralis* and ‘*N. mucosa* var. *heidelbergensis*’ should therefore be consolidated into a single species group with the validly published name *N. oralis*.

We suggest that the initial species identification of members of the genus *Neisseria* should include: growth on media specific for *Neisseria*, such as LBVT.SNR medium for non-pathogenic *Neisseria* species and modified Thayer–Martin medium for the pathogens *N. meningitidis* and *N. gonorrhoeae* (Knapp & Hook, 1988); colony description, for example transparent or opaque, non-haemolytic, mucoid convex colonies approximately 1–5 mm in diameter; Gram-negative; and oxidase positive. Microscopic morphology is useful, although there is variation within the genus *Neisseria* as some members are coccoid and some are bacilliform. Sequencing of the 16S rRNA or 23S rRNA genes can determine whether an isolate is a member of the genus *Neisseria*, but an analysis of multiples genes, either rMLST or cgMLST, is necessary to identify the sequence clusters that correspond to the individual species within the genus.

Analyses of phenotypic characteristics are problematic in the genus *Neisseria*, as with many other genera, due to high levels of variation among and within species. For example, in the *N. oralis* description (Wolfgang *et al.*, 2013), two of the five strains examined (8261 and F0314) exhibited β-galactosidase activity when analysed using API NH and API ZYM tests. The presence of β-galactosidase activity is considered indicative of *N. lactamica*, as this was thought to be the only species of the genus *Neisseria* able to ferment lactose; however, the *N. oralis* isolate F0314, for which there is a genome sequence available, had intact *lacY* and *lacZ* genes (designated NEIS2199 and NEIS2200, respectively, at http://pubmlst.org/neisseria/), necessary for lactose fermentation. The detection of β-galactosidase activity in some *N. oralis* strains indicates that this is not a reliable test to differentiate *N. lactamica* from other species and suggests that some isolates identified previously as representing *N. lactamica* may in fact be members of *N. oralis*. The *lacZ* and *lacY* gene variants from isolate F0314, NEIS2199 allele 3 and NEIS2200 allele 2, respectively, and 16 *lacZ* and nine *lacY* alleles from *N. lactamica* are available from http://pubmlst.org/neisseria/.

Classification systems can only work well if strains are accurately and comprehensively characterized (Tindall *et al.*, 2010). Phylogenies generated from 16S rRNA gene sequences are inadequate to differentiate *Neisseria* taxonomically, and molecular characterization cannot be based on these data alone. A much greater degree of resolution can be obtained by indexing variation in multiple protein coding genes, as recommended for prokaryote characterization (Stackebrandt *et al.*, 2002). It is also important that all relevant strains are used to determine relationships, not just the type strains of species. Analyses of WGS data and subsets thereof, such as rMLST and cgMLST, have the potential to replace the polyphasic approach to bacterial taxonomy, which is both labour-intensive and complex, requiring specialized skills and laboratories (Stackebrandt & Ebers, 2006). DNA–DNA hybridization, for example, is not as informative as rMLST or cgMLST, as it can only be used to compare genomes indirectly, whereas these analyses allow direct comparisons. It has been shown that cellular fatty acid analysis is not useful in distinguishing among species within the genus (Wolfgang *et al.*, 2013), and biochemical tests may not be informative as results can be variable and their analysis subjective. As WGS determination has become relatively inexpensive and rapid, there is no need to rely on small gene fragments deposited in uncurated databases to aid bacterial taxonomic differentiation. Comprehensive, high-quality reference datasets, obtained from curated databases such as those hosted at PubMLST.org, are now publicly available, with sequences from any number of genes and genomes easily and rapidly aligned. We recommend that at least two strains for each novel species are deposited in culture collections and that their genome sequences are available in public databases.
